# Identification of Energy Metabolism-Related Gene Signatures From scRNA-Seq Data to Predict the Prognosis of Liver Cancer Patients

**DOI:** 10.3389/fcell.2022.858336

**Published:** 2022-05-04

**Authors:** Boyang Xu, Ziqi Peng, Yue An, Guanyu Yan, Xue Yao, Lin Guan, Mingjun Sun

**Affiliations:** ^1^ Department of Gastroenterology, The First Affiliated Hospital of China Medical University, Shenyang, China; ^2^ Department of Breast Surgery, The First Affiliated Hospital of China Medical University, Shenyang, China; ^3^ Department of Endoscopy, The First Hospital of China Medical University, Shenyang, China; ^4^ Department of Surgical Oncology, The First Hospital of China Medical University, Shenyang, China

**Keywords:** ScRNA-seq, liver cancer, energy metabolism, molecular subtypes, immune function

## Abstract

The increasingly common usage of single-cell sequencing in cancer research enables analysis of tumor development mechanisms from a wider range of perspectives. Metabolic disorders are closely associated with liver cancer development. In recent years, liver cancer has been evaluated from different perspectives and classified into different subtypes to improve targeted treatment strategies. Here, we performed an analysis of liver cancer from the perspective of energy metabolism based on single-cell sequencing data. Single-cell and bulk sequencing data of liver cancer patients were obtained from GEO and TCGA/ICGC databases, respectively. Using the Seurat R package and protocols such as consensus clustering analysis, genes associated with energy metabolism in liver cancer were identified and validated. An energy metabolism-related score (EM score) was established based on five identified genes. Finally, the sensitivity of patients in different scoring groups to different chemotherapeutic agents and immune checkpoint inhibitors was analyzed. Tumor cells from liver cancer patients were found to divide into nine clusters, with cluster 4 having the highest energy metabolism score. Based on the marker genes of this cluster and TCGA database data, the five most stable key genes (ADH4, AKR1B10, CEBPZOS, ENO1, and FOXN2) were identified as energy metabolism-related genes in liver cancer. In addition, drug sensitivity analysis showed that patients in the low EM score group were more sensitive to immune checkpoint inhibitors and chemotherapeutic agents AICAR, metformin, and methotrexate.

## Introduction

The new incidence of liver cancer accounted for 4.7% of all malignant tumors, sixth among all malignancies, and deaths due to liver cancer were nearly 830,000, accounting for 8.3% of all cancer deaths (Siegel et al., 2020) according to the global cancer data released by WHO International Agency for Research on Cancer (IARC) in 2020. Since liver tumors are highly heterogeneous, a variety of molecular typing schemes for liver cancer based on gene mutations and the clinical characteristics of liver cancer have been developed previously. For example, Boyault constructed a G1-G6 typing scheme related to the clinical and genetic characteristics of liver cancer based on P53, PIK3CA, hepatitis B virus (HBV) copy numbers and other indicators (Boyault et al., 2007). Hoshida constructed an S1-S3 typing scheme according to the expression levels of MyC, EpCAM, and other genes (Hoshida et al., 2009). There are also differences in prognosis and sensitivity to drug treatment between liver cancer subtypes, which may assist the development of patient-specific treatment strategies.

Tumor metabolism has received increasing attention from researchers in recent years. Reprogramming of energy metabolism is a characteristic feature of tumor cells that promotes rapid cell growth and proliferation. Tumor cells actively ingest glucose through an unusual “anaerobic glycolysis” process (known as the Warburg effect), which not only provides energy for the tumor cell but also allows intermediates to enter side pathways of anabolism to maintain the *de novo* synthesis of nucleotides, lipids, and amino acids required for cell proliferation (Peter et al., 2019). In addition, organic acids produced due to the Warburg effect cause acidification of the extracellular environment and thus promote ECM degradation and facilitate invasion of tumor cells, resulting in poor prognosis of patients (Jianrong, 2019). However, studies have shown that tumor cells can inhibit the function of tumor-infiltrating immune cells through competitive uptake of nutrients, and metabolites such as lactic acid and cholesterol can further inhibit the function of immune cells, resulting in immune escape (Siska et al., 2020). The liver is the largest metabolic organ in the human body, and metabolic abnormalities are thus closely associated with the occurrence and development of liver cancer. Research on metabolism has also provided new directions for liver cancer diagnosis and treatment (Satriano et al., 2019).

scRNA-seq has been widely used to identify previously unknown tumor subtypes in recent years (Timour and James, 2017). Here, single-cell sequencing data were used to classify liver cancer to obtain genes related to energy metabolism. Finally, a liver cancer cell energy metabolism score (EM score) was constructed to accurately predict the prognosis of patients.

## Materials and Methods

### Data Collection

Single-cell RNA sequencing data (scRNA-seq) of liver cancer patients were downloaded from the GEO database (https://www.ncbi.nlm.nih.gov/geo/, accession number GSE146115). Avereps function in R package Limma was used to average the expression data of the same gene names. Data for liver cancer patients were obtained from the TCGA database (https://portal.gdc.cancer.gov/), including the transcriptome data [RNA-seq; in fragments per kilobase million (FPKM)] and other relevant clinical information. RNA-seq and related clinical data of liver cancer patients were obtained from the ICGC database (https://dcc.icgc.org/), and used as the validation set.

### Identification of Liver Cancer Cell Subtypes From scRNA-Seq Data

The scRNA-seq data were analyzed using the Seurat R package. Samples with mitochondrial gene percentages greater than five were excluded. The NormalizeData function was used to standardize the data and extract 1,500 genes with a high coefficient of variation between cells. PCAP was then performed, and *p* values of each principal component were computed. Principal components with *p* values less than 0.05 were selected for the subsequent t-SNE analysis, where cells were divided into different clusters. The SingleR R package was used to annotate the cell types of the obtained cell clusters, and a liver cell expression matrix was extracted for further analysis (Aran et al., 2019). The Seurat package was used to perform the same analysis on the liver cell expression matrix, and the liver cancer cells were divided into different clusters. To analyze the differences between different cluster genes, the genes with *p* values less than 0.05, and log2 |FC| greater than 1 were considered markers of the cluster. Time-series (trajectory) analysis of liver cancer cells was performed using monocle R package to determine the differentiation direction of liver cancer cells.

### Determination of Energy Metabolism Score in Different Subtypes of Liver Cancer Cells

Annotated gene information on human energy metabolism pathways was downloaded from the Reactome database (https://reactome.org/). ssGSEA analysis was then performed on the scRNA-seq of liver cancer cells using the annotated gene information, and the scores of each cell in different energy metabolism pathways were obtained. Heat maps were constructed using the pheatmap R package, and cluster assignments were added to reflect the metabolic status of different clusters on the heatmaps. Marker genes of the cluster with the highest metabolic score were then selected for further analysis.

### Identification of Key Genes From the TCGA-LIHC RNA-Seq Data

Differential expression analysis of marker genes (Normal tissue vs. Tumor tissue) in the TCGA-LIHC RNA-seq data was performed using the following criteria to determine differentially expressed genes: *p* < 0.05, and log2 |FC|>1. A univariate Cox analysis was also performed to analyze prognosis-associated marker genes. The intersection of the two gene lists obtained from these procedures was determined, and genes with expression levels inconsistent with the prognosis were removed. TCGA FPKM data were converted to TPM format, and the ConsensusclusterPlus package was used to perform consistent cluster analysis for genes within the intersection. TCGA-LIHC patients were thus divided into different clusters, and K-M survival analysis was used to determine the difference in survival times between the different clusters. Subsequently, 1,000 times lasso analyses were conducted to screen out the most stable genes as key genes, and consistency cluster analysis was conducted once again for the selected genes to determine the survival differences among different clusters.

### Validation of Selected Key Genes

PCA was applied to verify the results of the consistency analysis and demonstrate that the identified key genes corresponded to different groups of liver cancer patients. Gene annotations of 25 metabolism-related pathways in the KEGG database (https://www.genome.jp/kegg/) were downloaded, and ssGSEA was used to calculate TCGA-LIHC patient-related scores to analyze the different metabolic pathway scores between clusters. Then, liver cancer patient data was selected from the ICGC database to verify the key gene-based grouping and prognostic correlation.

### Determination of High and Low Score Groups Based on Energy Metabolism Score Calculation

According to the previous typing results, if the typing value was found to increase with decreasing gene expression, the gene was classified as “signal A” gene. Vice versa, if gene expression was found to increase with increasing typing values, the gene was classified as “signal B” gene. PCA was used to calculate the energy metabolism score (EM score) of each sample using the following equation:

EM Score=∑PC1A−∑PC1B

Used the SURv_cutpoint function to filter the best truncation value, and the patients were divided into high-and low-score groups. The difference in survival between the high-and low-score groups was subsequently analyzed.

### Analysis of the Association Between Energy Metabolism Score and Immune Cells/Functions

The annotated gene information for 16 types of immune cells and 13 types of immune functions was downloaded from the GSEA database (http://www.gsea-msigdb.org/). The scores of immune cells and immune function of patients in the different evaluation groups were calculated using ssGSEA, and the differences in ssGSEA scores between the high and low EM score groups were analyzed. Additionally, immune cell and stromal cell scores in TCGA-LIHC patients were calculated using the Estimate package, and the correlation between the EM score and the estimated immune score was calculated using Spearman’s correlation coefficient. Expression data of immune checkpoint genes were extracted from patients, and the differences in immune checkpoint levels between the high-and low-score groups were calculated. Statistical significance was set at *p* < 0.05.

### Determination of the Sensitivity of Patients in High and Low Groups to Tumor Treatment Drugs

pRRophetic is an R package created from gene expression and drug sensitivity data of cell lines from the Cancer Genome Project to predict clinical chemotherapy response from the perspective of tumor gene expression levels (Geeleher et al., 2017). Sensitivity to metabolism-related chemotherapeutic agents in patients with liver cancer was assessed using this package, and differences in sensitivity between the groups were analyzed (*p* < 0.05 was considered statistically significant). The sensitivity differences of TCGA-LIHC patients to PD-1 and CTLA4 inhibitors were analyzed using immunotherapy sensitivity data from the Cancer Immunochromatographic Database (TCIA) (https://tcia.at/).

## Results

### Data Collection

The scRNA-seq data used in this study was obtained from 3,200 cells of four liver cancer patients. Bulk RNA-Seq was obtained from TCGA and ICGC databases. The TCGA database contains 374 tumor samples and 50 paracancer normal tissue samples. The ICGC database contains gene expression data and clinical information for 231 patients with liver cancer. Patient information related to TCGA and ICGC is shown in Table 1.

**TABLE 1:** Clinical characteristics of the liver cancer patients used in this study.

	TCGA	ICGC
Survival status		
Alive	245	189
Dead	132	42
Gender		
Male	255	170
Female	122	61
Age(median, range)	61 (16–90)	69 (31–89)
Stage		
I	175	36
II	87	105
III	86	71
IV	5	19
Uknown	24	0
Grade		
1	55	NA
2	180	NA
3	124	NA
4	13	NA
Uknown	5	NA

### Identification of Liver Cancer Cell Subtypes

**FIGURE 1:**
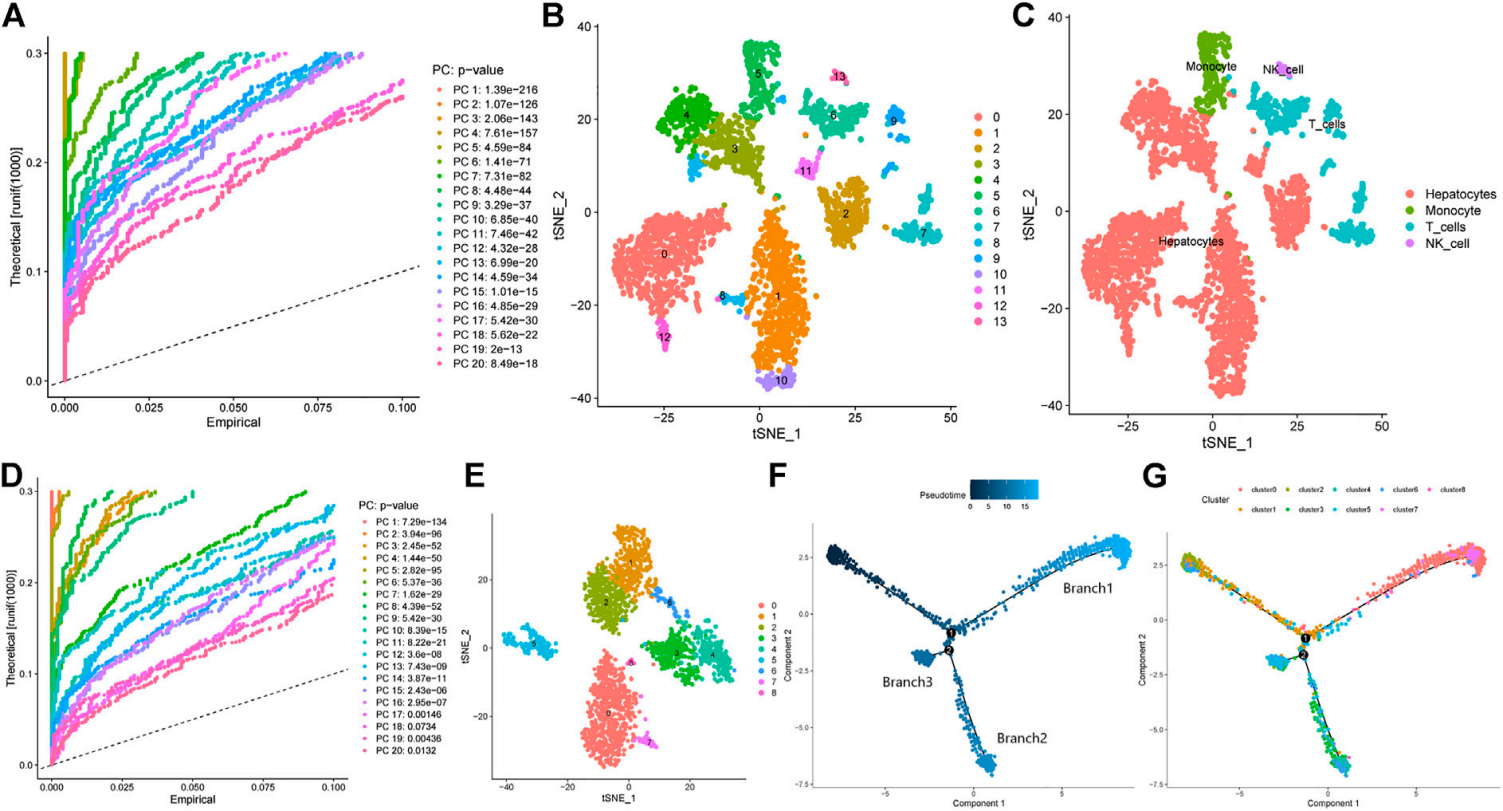
Screening of high-energy metabolism hepatocellular carcinoma subtypes using scRNA-seq data. **(A)** Principal component analysis of all cells and *p*-value of each principal component. **(B)** tSNE algorithm divides cells into 14 clusters. **(C)** Cell types of different clusters. **(D)** Principal component analysis of hepatocellular carcinoma cells and the *p*-value of each principal component. **(E)** tSNE algorithm divides hepatocellular carcinoma cells into 9 clusters. **(F)** Proposed time series analysis of hepatocellular carcinoma cells. **(G)** Proposed time series analysis of different clusters of hepatocellular carcinoma cells.

The Seurat R package was used to analyze scRNA-seq liver cancer cells. The FindVariableFeatures function of Seurat was used to extract the top 1,500 genes with the largest coefficients of variation for PCA. The *p* values of the top 20 principal components are shown in Figure 1A. The identified top 20 principal components were incorporated into the t-SNE analysis, where 3,200 cells were divided into 14 clusters (cluster 0–13, Figure 1B). Subsequently, the differentially expressed genes within each cluster were identified (*p* < 0.05). These differentially expressed genes were defined as marker genes for the corresponding clusters (Supplementary Table S1). To investigate the characteristics of the cells within each cluster, cell type annotations were obtained using the SingleR R package. The results revealed 14 clusters which included four cell types, with cells in cluster 5 containing monocytes, cells in clusters 6, 7, and 9 T cells, cells in cluster 13 NK cells, and cells in the remaining clusters hepatocytes (Figure 1C). GSEA analysis of differentially expressed genes yielded higher and lower metabolism- and immune system-related scores in hepatocyte cells, respectively. On the other hand, the opposite was observed for immune cells (Supplementary Figure S1), which highlighted changes in metabolism of liver cancer cells. To further explore liver cancer cell subtypes, we extracted the gene expression matrix of hepatocyte cells (in total 2,302 cells), and performed the same analysis as before using the Seurat package. PCA revealed a total of 15 principal components with *p*-values less than 0.05 (Figure 1D). These principal components were incorporated into the t-SNE analysis (Figure 1E). Accordingly, the hepatocytes were divided into nine clusters (0–8), and the marker genes of each cluster are shown in Supplementary Table S2. The monocle R package was used to perform a time-series analysis of the these hepatocytes to explore their differentiation directions. The results showed that hepatocytes gradually followed three differentiation directions, which are termed Branch 1, Branch 2 and Branch 3 (Figure 1F). The differentiation directions of hepatocytes were combined with the cluster information, and calculated for the major components in different Branch clusters. Figure 1G shows that the cells that account for the major component in Branch 1 stemmed from cluster 0 (688/809), those in Branch2 from cluster 4 (352/537), and those in Branch 3 mainly from cluster 5 (138/214).

### Identification of Hepatocyte Cancer Cell Subtypes With High Energy Metabolism

**FIGURE 2:**
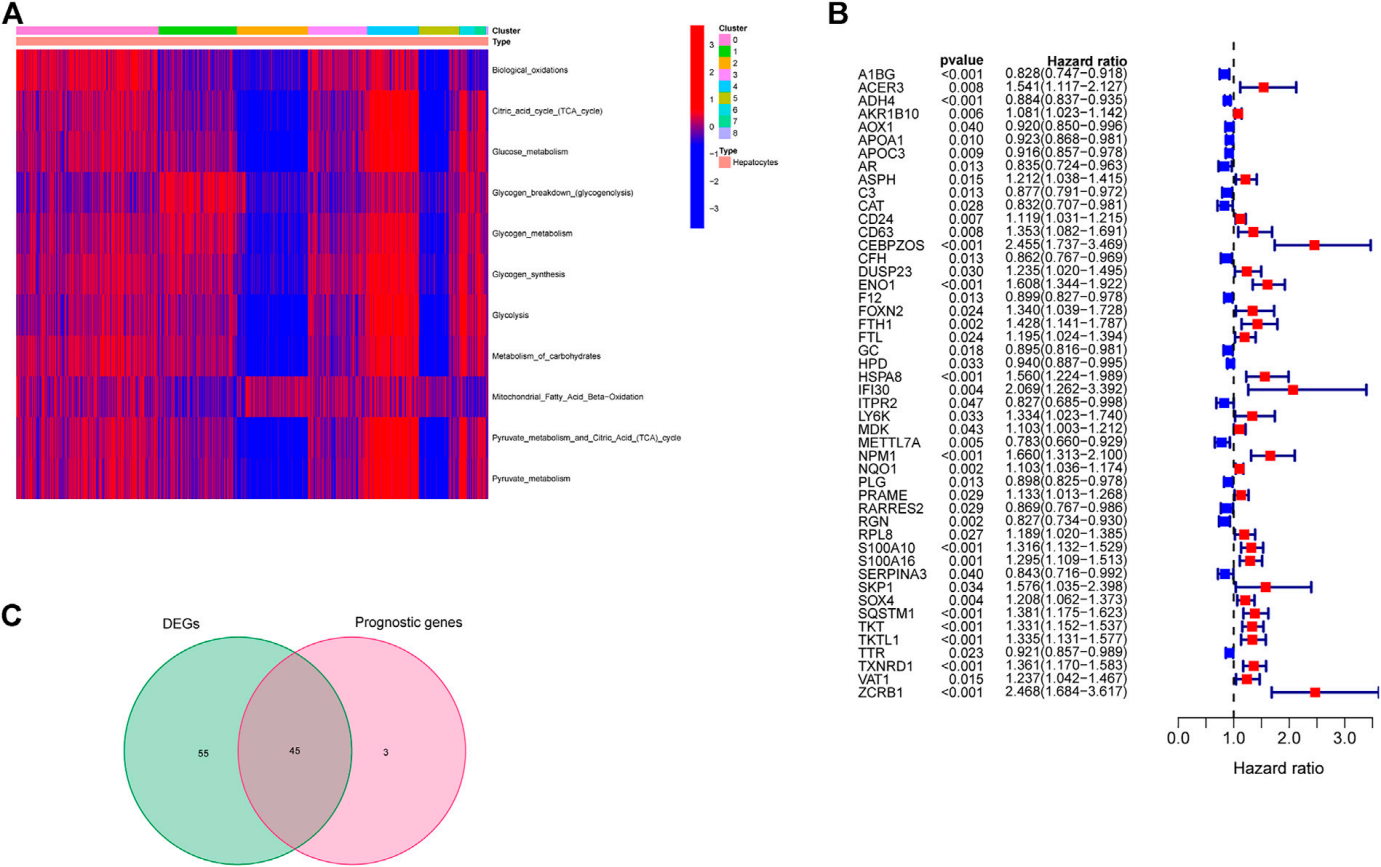
Screening of genes related to high energy metabolism. **(A)** Energy metabolism scores of differentcluster hepatocellular carcinoma cells. **(B)** Marker genes associated with survival in high energy metabolism cluster hepatocellular carcinoma cells. **(C)** Intersection of prognosis-related marker genes and differentially expressed marker genes.

Based on the annotated genes of the 11 energy metabolism-related pathways downloaded from the Reactome database (see Table 2 for details), ssGSEA analysis was performed to obtain the score of each cell in these pathways. Combining this scoring and cluster information of each cell, the pheatmap R package was used to plot a heat map that shows the scoring status of different clusters. As shown in Figure 2A, clusters 4 and 5 yielded the highest and lowest energy metabolism related scores, respectively. Cluster 0, the largest cluster, yielded a moderate metabolism score. Taken together with the results of the time-series analysis in the previous section, liver cancer cells were concluded to eventually differentiate into three different states of subtypes in terms of energy metabolism: high, medium and low, with Branch 2 showing the highest level of energy metabolism.

**TABLE 2:** Metabolic pathways.

Metabolic pathway	PathwayID	Gene count
Biological oxidations	R-HSA-211859	221
Citric acid cycle (TCA cycle)	R-HSA-71403	22
Glucose metabolism	R-HSA-70326	92
Glycogen breakdown (glycogenolysis)	R-HSA-70221	15
Glycogen metabolism	R-HSA-8982491	27
Glycogen synthesis	R-HSA-3322077	16
Glycolysis	R-HSA-70171	72
Metabolism of carbohydrates	R-HSA-71387	292
Mitochondrial fatty acid beta-oxidation	R-HSA-77289	38
Pyruvate metabolism	R-HSA-70268	31
Pyruvate metabolism and citric acid (TCA) cycle	R-HSA-71406	55

### Identification of a TCGA-LIHC High-Energy Metabolic Subtype

Marker gene expression data of cluster 4 were extracted from TCGA-LIHC data, and analyzed to determine differentially expressed genes between cancerous and normal tissues. A total of 101 differentially expressed genes were obtained. Univariate Cox regression analysis was performed in combination with the overall survival time of patients, and a total of 48 genes associated with OS were obtained (Figure 2B). The intersection of differentially expressed and prognosis-related genes was determined. A gene was excluded if its expression level in the tumor was found to be inconsistent with prognosis as described in Methods section. As a result, a total of 45 genes were identified from the initial intersection list (Figure 2C).

**FIGURE 3:**
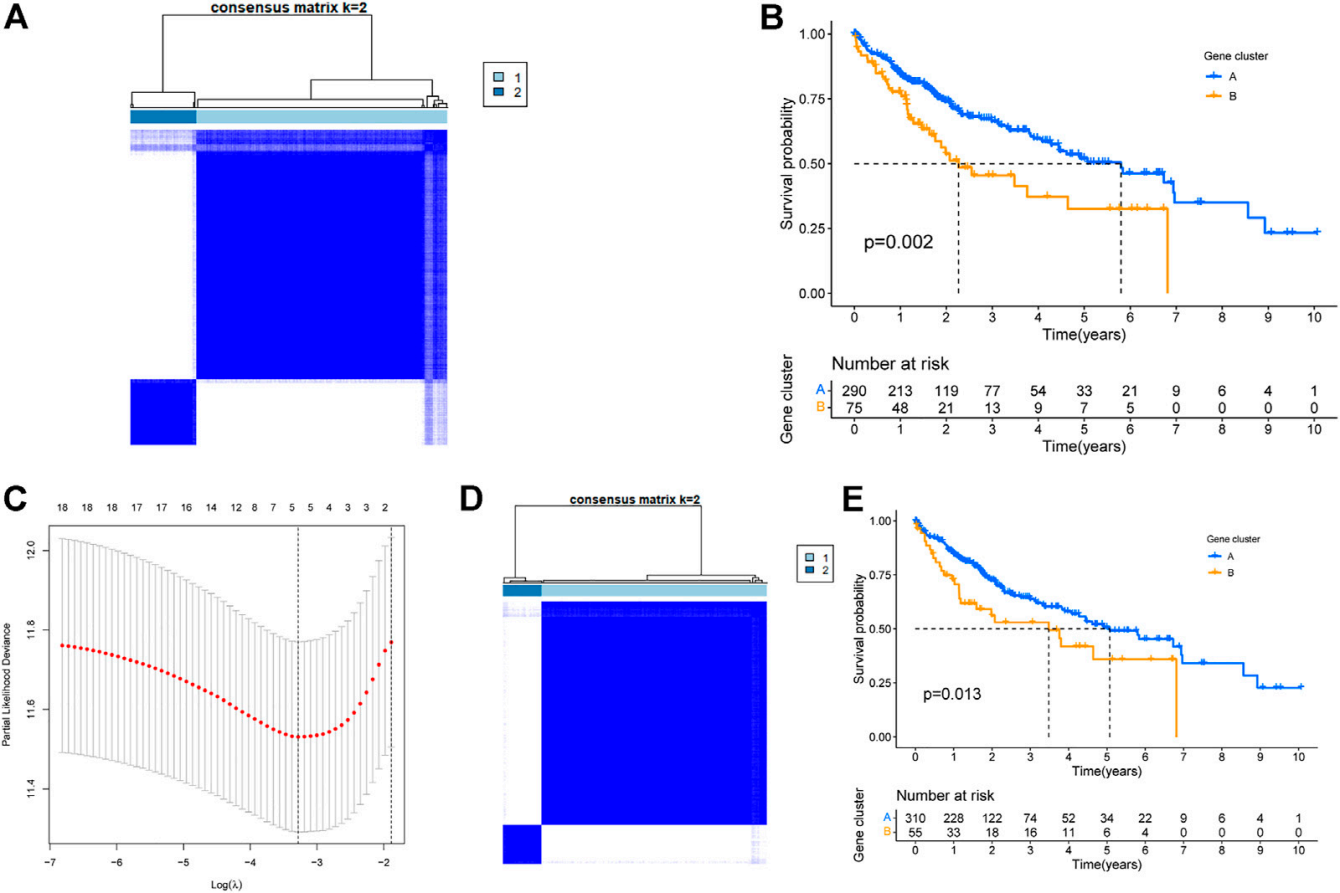
Identification of TCGA-LIHC energy metabolism subtypes. **(A)**Typing of TCGA-LIHC based on 45 intersecting genes. **(B)** Survival of TCGA-LIHC patients within different subtypes based on 45 intersecting genes. **(C)** Lasso analysis to screen for the most stable energy metabolism-related genes. **(D)** TCGA-LIHC typing based on the five most stable energy metabolism-related genes. **(E)** Survival of TCGA-LIHC patients within different subtypes based on five key genes.

Based on the expression data of these 45 genes, a clustering analyses was performed. Based on the cumulative distribution function (CDF), we chose k = 2 as the optimal clustering parameter that yielded consistent results, and thus divided TCGA-LIHC patients into two clusters named cluster A and cluster B (Figure 3A). Survival analysis revealed that the OS of patients in clusters A and B were significantly different (*p* = 0.002, Figure 3B). Thus, we inferred that these 45 genes could influence the OS of liver cancer patients by affecting their metabolic status. Furthermore, 1000 lasso analysis also identified five genes (Figure 3C): *ADH4*, *AKR1B10*, *CEBPZOS*, *ENO1*, and *FOXN2*, as the most stable prognosis-related genes associated with energy metabolism in liver cancer. The correlation results among the five genes were shown in Supplementary Table S5 and Supplementary Figure S2 (Correlations between genes greater than 0.2 were shown in Supplementary Figure S2). The immunohistochemical results of the expression of the above five genes in liver cancer and normal tissues in HPA database were shown in Supplementary Figure S3.

To explore the significance of these five genes, another clustering analysis was performed to divide TCGA-LIHC patients into two clusters, cluster A and cluster B (Figure 3D). Survival analysis revealed that the OS of cluster B was significantly lower than that of cluster A, and the difference was statistically significant (*p* = 0.013, Figure 3E).

### Validation of the Identified Key Genes

**FIGURE 4:**
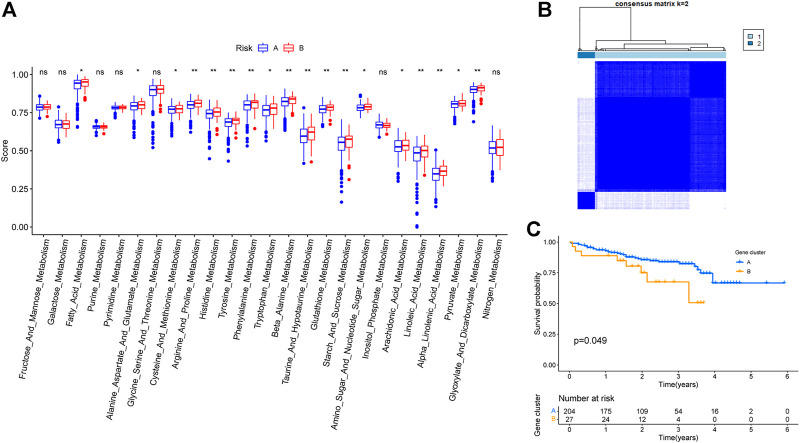
Validation of TCGA-LIHC typing results based on 5 key genes. **(A)** Differences in metabolism-related pathway scores among different subtypes. **(B)** Staging results of patients with hepatocellular carcinoma in ICGC database. **(C)** Survival of patients within different subtypes in the ICGC database.

ssGSEA results revealed that multiple higher metabolic pathway scores were higher in cluster B compared to cluster A (Figure 4A). The same analysis was performed on the liver cancer data obtained from the ICGC database to test whether choice of the dataset affected our conclusions. Based on ICGC data, the same five genes were able to classify liver cancer patients into two clusters (Figure 4B), and the survival analysis between the two groups showed a statistically significant difference with respect to OS (Figure 4C).

### Determination of Energy Metabolism Scores

**FIGURE 5:**
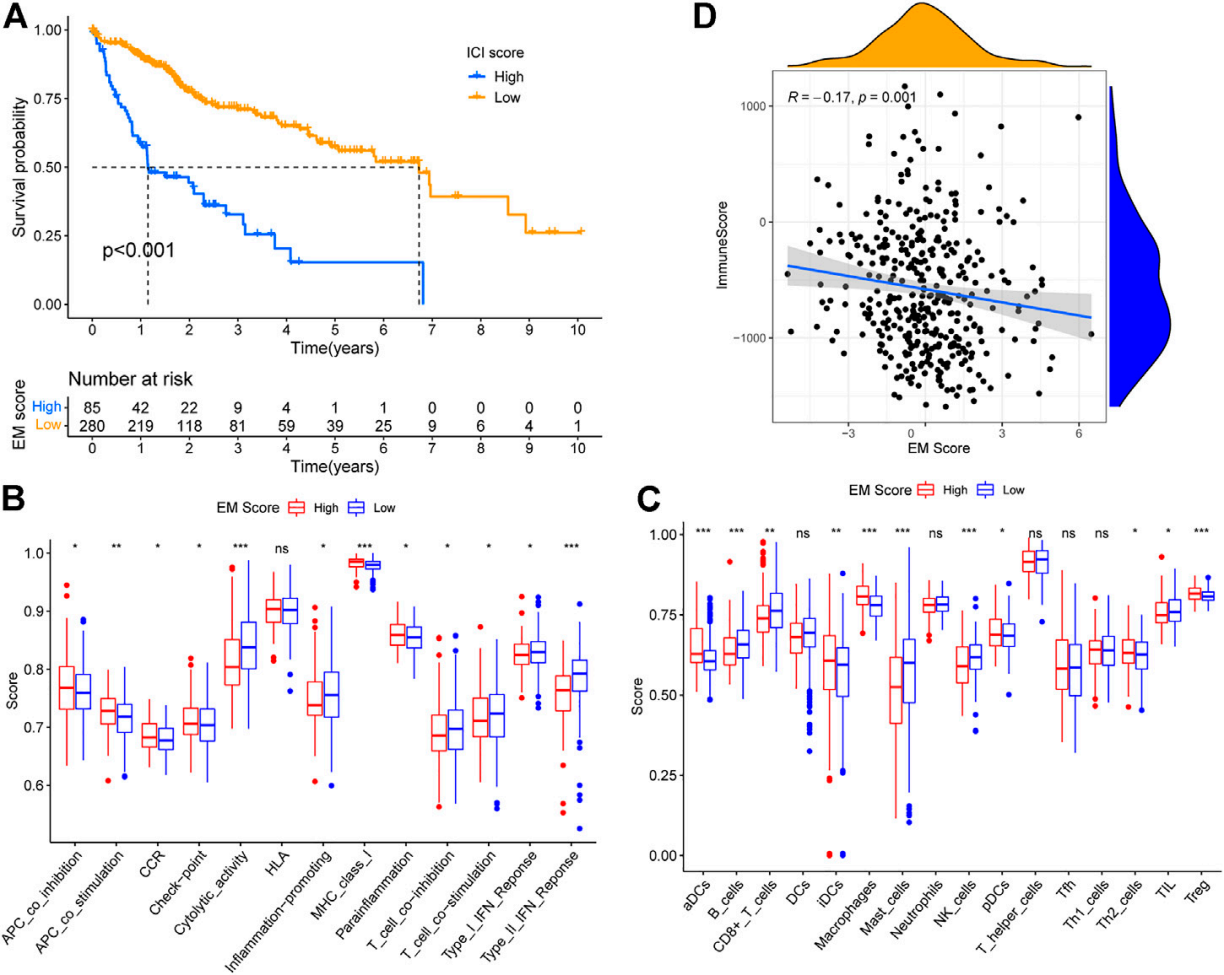
Construction of EM score and its relationship with immunity. **(A)** Survival of patients within different EM score groups. **(B)** Correlation between EM score and Estimate ImmuneScore. **(C)** Differences in immune function scores of patients within different EM score groups. **(D)** Differences in immune cell scores of patients within different EM score groups.

To determine an energy metabolism score for TCGA-LIHC patients, we used principal component analysis (PCA) to calculate PC1 for genes within gene tags A and B, and calculated the sum of PC1 for both tags A and B (sPC1A and sPC1B), respectively. Subsequently, the difference between sPC1A and sPC1B was used as the energy metabolism score (EM score). Using the best cut-off value, TCGA cohort patients were divided into groups of high or low EM scores. Survival analysis showed that patients in the high score group had significantly worse survival than those in the low score group (*p* < 0.001) (Figure 5A). Thus, the EM score was successfully used to accurately classify patients based on overall survival.

### The Relationship Between Energy Metabolism Score and Immunity

**FIGURE 6:**
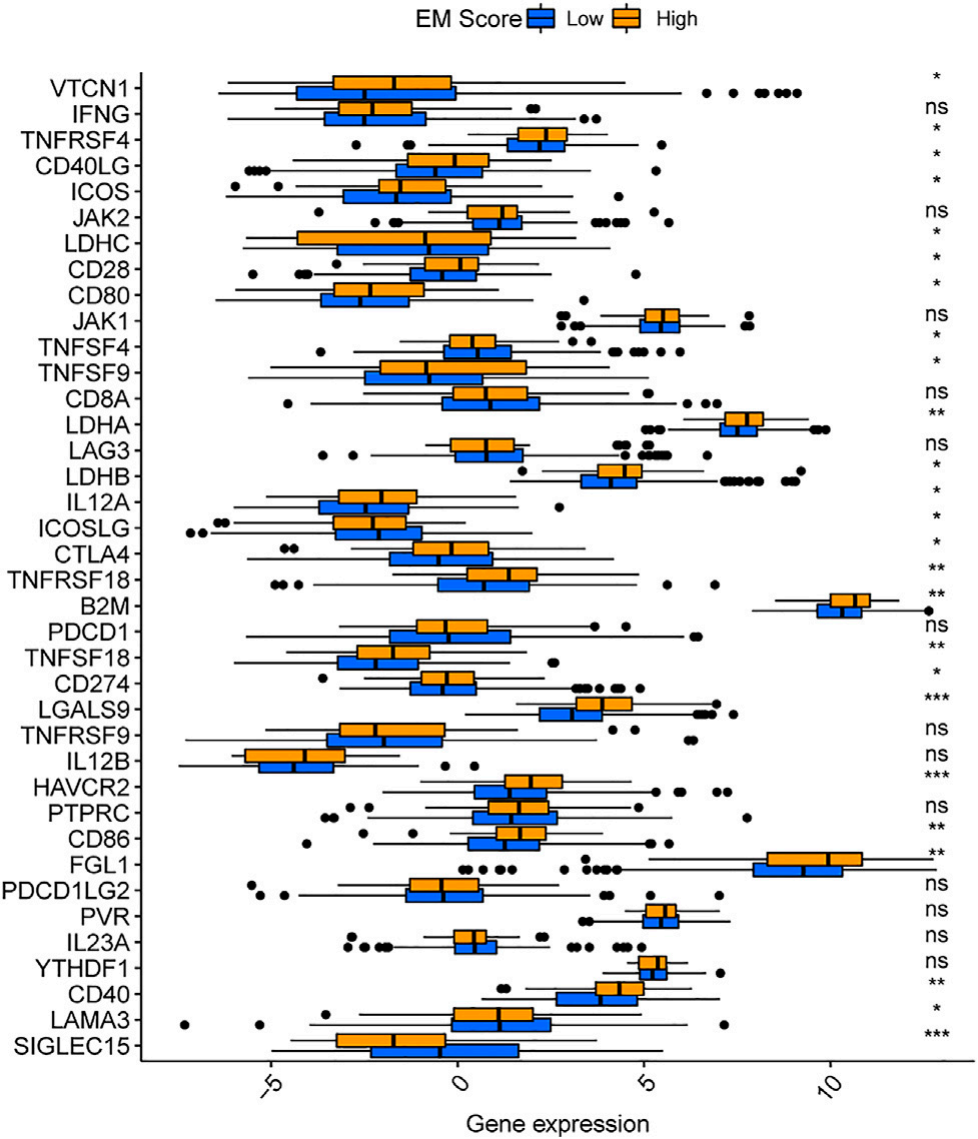
Differences in immune checkpoint levels between different EM score groups.

There is substantial evidence in literature on competition between tumor and immune system cells for nutrients, and inhibition of immune cell function by tumor cell metabolites (Xing et al., 2015). To explore the relationship between tumor energy metabolism and immunity in liver cancer, we analyzed 16 immune cell and 13 immune function scores in TCGA-LIHC patients by ssGSEA, and used the Wilcoxon test to determine the significance of the differences in scores of high and low EM Score groups. A total of 11 immune cell and 12 immune function scores were found to differ between the high and low EM Score groups (Figures 5B,C), with the majority being higher in the low EM Score group. Estimate analysis showed a decreasing trend of ImmuneScore (R = -0.17, *p* = 0.001) in TCGA-LIHC patients with increasing EM Score, and the results were statistically significant (Figure 5D). In addition, 25 of the 38 immune checkpoints differed between the high and low EM score groups, with most being highly expressed in the high EM score group (Figure 6). These results suggest that EM score can be used as an indicator of an influence on immunity in liver cancer patients.

### The Role of Energy Metabolism Score in Predicting Drug Sensitivity in Cancer Therapy

**FIGURE 7:**
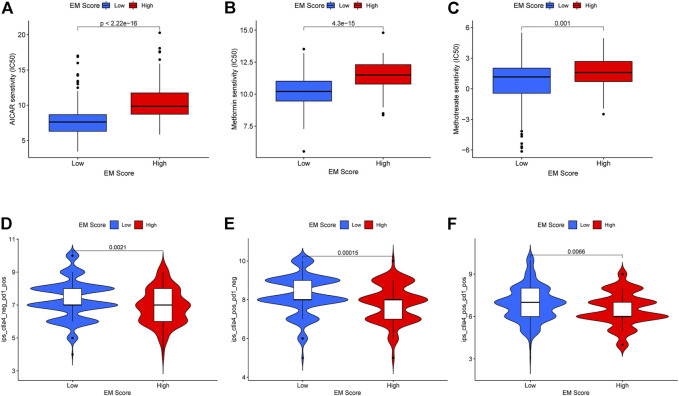
Relationship between EM score and sensitivity of chemotherapy drugs and immune checkpoint inhibitors. **(A–C)** Differences in sensitivity to chemotherapeutic agents AICAR, Metformin and Methotrexate among patients in different EM score groups. **(D–F)** Differences in sensitivity to PD1 inhibitors, CTLA-4 inhibitors and the combination of these two inhibitors in different EM score groups.

Using the pRRophetic R package, the sensitivity (IC50) of patients to 138 chemotherapeutic agents, including AICAR, metformin, and other metabolism-related anticancer drugs, were determined. Accordingly, liver cancer patients in the low EM score group had higher sensitivity (lower IC50) to three metabolism-related anticancer drugs: AICAR (*p* = 2.22e-16), metformin (*p* = 4.3e-15), and methotrexate (*p* = 0.001) (Figures 7A–C). The TICA database data showed that liver cancer patients within the low EM score group had higher sensitivity to CTLA4 inhibitors (*p* = 0.00015), PD-1 inhibitors (*p* = 0.0021), and PD-1 inhibitors combined with CTLA4 inhibitors (*p* = 0.0066) (Figures 7D–F). Taken together, these data suggest that the EM score may be associated with response to chemotherapeutic agents in liver cancer patients.

## Discussion

Liver cancer is a disease that seriously threatens human health. The disease ranked sixth in new cases and third in deaths among all malignant tumors in 2020. Therefore, in-depth investigations of the mechanism of liver cancer development has been prioritized by cancer researchers. The liver is the largest metabolic organ in the human body, and alterations in its metabolic profile are often closely related to the development of a primary tumor (Pope et al., 2019). Liver cancer cells in a hypermetabolic state are not only able to compete with normal cells for nutrients, but their metabolites can also affect the function of immune cells and promote tumor metastasis (Hanahan and Weinberg, 2011; Wu, 2017). Here, we focused on the metabolic status of different subtypes of liver cancer, and constructed and validated an energy metabolism (EM) score to quantify the metabolic status of liver cancer patients based on scRNA sequencing, TCGA-LIHC, and ICGC database data. Our findings showed that the EM score could be used to accurately predict the prognosis of liver cancer patients, and has potential to assist the selection of drugs for patient-specific treatment options.

Many studies have previously shown that the development of liver cancer is accompanied by disturbances in energy metabolism, which not only suppresses immune response to tumors, but also promotes tissue invasion and metastasis. Therefore, it is highly relevant to study the genes related to energy metabolism in liver cancer, and developed a score to characterize the status of energy metabolism in liver cancer patients. Here, we obtained liver cancer single-cell sequencing data from the GEO database, clustered and annotated the cells, and used ssGSEA to identify a subgroup of liver cancer cells with a high energy metabolism level. Based on the marker genes in this subgroup, we used univariate and Lasso Cox regression analysis to screen the TCGA database data for the most accurate prognostic biomarkers to establish an energy metabolism-related gene signature. As a result, we obtained a signature containing five genes: *ADH4*, *AKR1B10*, *CEBPZOS*, *ENO1*, and *FOXN2*. *ADH4* encodes the class II ethanol dehydrogenase 4 subunit, which is a dimer consisting of two subunits with high oxidative activity towards long-chain fatty alcohols and aromatic alcohols. *ADH4* has been associated with various disorders such as alcohol dependence (Chowdhury et al., 2020). Differential expression of *ADH4* between tumor and normal tissues in patients with non-small cell lung cancer has been observed, and can be used as a prognostic marker in patients with esophageal cancer (Peng et al., 2018). Wei et al. showed that *ADH4* was expressed at lower levels in the tumor tissues of liver cancer patients, and patients with low *ADH4* expression levels had significantly shorter survival times (Wei et al., 2012).

*AKR1B10* encodes a member of the aldehyde/ketone reductase superfamily, which is overexpressed in various solid tumors, and a potential diagnostic marker for tumors (Endo et al., 2021). In breast cancer, this gene enhances fatty acid utilization by tumor cells and activates ERK, Wnt, and other pathways to promote metastasis (Qu et al., 2020). In liver cancer, *AKR1B10* mediates the proliferation of liver cancer cells through sphingosine-1-phosphate (Jin et al., 2016). In addition, *AKR1B10* induces cellular resistance to erythromycin and nortriptyline by reducing the C13 ketone moiety, which leads to poor patient prognosis (Zhong et al., 2011).

*CEBPZOS* is the antisense chain of CEBPZ, and is expressed in various tissues. Only several studies have been conducted on *CEBPZOS*, yet it has been shown to be a novel mitochondrial protein (Hung et al., 2014). Due to their key role in intracellular energy generation, mitochondria have a great impact on cell growth, apoptosis, and maintenance of redox homeostasis. In tumors, oxidative phosphorylation of the mitochondria is very active, and mitochondria are thus primary sites where the Warburg effect is strong (Wallace, 2012). The role of CEBPZOS as a mitochondrial protein may also affect the energy metabolism of tumor cells in liver cancer, and thereby affect tumor development as well.

*ENO1* encodes α-enolase, which is one of the three enolase isozymes found in mammals (Zhang et al., 2020). *ENO1* is overexpressed in a variety of GI tumors, including colon, pancreatic, and gastric cancers (Hang et al., 2018), and promotes tumor development (Cheng et al., 2019; Xu et al., 2019). In addition, *ENO1* is highly expressed in liver cancer, promotes glucose uptake and lactate production by tumor cells, and is involved in tumor cell division, proliferation, apoptosis, metastasis, immunomodulation, and chemoresistance (Qiao et al., 2018; Jiang et al., 2020).

*FOXN2* plays an important role in the development of several tumors (Ye and Duan, 2019), including breast and cervical cancers (Cui et al., 2019). *FOXN2* has been shown to be associated with tumor cell proliferation and invasion in liver cancer (Liu et al., 2021).

Based on these five genes, patients were divided into two groups, cluster A and cluster B. The prognosis of the two groups was found to be significantly different from each other. The energy metabolism of the patients was also analyzed using ssGSEA, and the results yielded significant differences in the energy metabolism-related pathway scores between the two groups. We conclude that these five genes are associated with the energy metabolism status of liver cancer patients, and can affect their overall survival. In addition, an external validation set from the ICGC database validated the accuracy of gene signatures consisting of these five energy metabolism-related genes for patient prognosis prediction.

Considering the variability in metabolic status among patients, it is important to quantify the status of energy metabolism of each patient. Identification of tumor subtypes based on different biomarkers has been used to improve the accuracy of patient prognosis for various tumors, such as breast cancer (Callari et al., 2016). Here, we used the above mentioned key genes as potential “subtype biomarkers,” and established an EM score to quantify the energy metabolism status of each sample. Survival analysis showed that higher EM scores were associated with lower survival rates.

Tumors are known to compete for glucose with immune system cells, and thus inhibit immune cell function. The glycolytic activities of tumor cells may also limit glucose consumption by immune cells, and thereby lead to immune escape (Fernández et al., 2019). In addition, metabolites produced by tumor cells can have profound effects on immune cells in the tumor microenvironment (TME). For example, aberrant glycolysis in tumor cells leads to the production of large amounts of lactic acid, resulting in an acidic TME. The low pH of the TME was shown to favor more aggressive tumor cells, and suppress immune response to tumors (Terrén et al., 2019). Arachidonic acid can also suppress immune responses by inhibiting Th1 differentiation, NK cell function, and T-cell activation (Pidgeon et al., 2007; Juman et al., 2013). Thus, we analyzed the differences in immune cell and immune function scores between the high and low EM score groups using ssGSEA to further explore the relationship between EM scores and immunity of liver cancer patients. In total, 11 immune cell and 12 immune function scores were found to differ between high and low EM score groups. Among them, B, T, and NK cells, which have anti-tumor effects, yielded higher scores in the low EM score group. However, the scores of immune checkpoints and immune functions with pro-cancer effects, such as CCR, were higher in the high EM score group. In addition, the results of the Estimate analysis showed that the immune and stromal scores of TCGA-LIHC patients decreased with increasing EM scores. In addition, the expression levels of most immune checkpoints were higher in liver cancer patients with high EM scores. These results suggest that the EM score reflects the status of the immune systems of liver cancer patients, with high EM scores representing lower immunity levels.

We also found that liver cancer patients in the low EM score group were more sensitive to three metabolism-related anticancer drugs, AICAR, metformin, and methotrexate, based on the results of the pRRophetic analysis. AICAR is an activator of AMP-activated protein kinase (AMPK) which can permeabilize cell membranes. Animal experiments have shown that AICAR significantly inhibits fatty acid and sterol synthesis in mouse hepatocytes (Gao et al., 2018), whereas cellular experiments have shown that AICAR inhibits the proliferation of liver cancer cells, and induces cell cycle arrest at the G1-S checkpoint (Cheng et al., 2014). Metformin is a drug related to glucose metabolism, and many studies have shown that metformin is not only effective against diabetes but also has therapeutic effects on a variety of tumors, including lung, pancreatic and breast cancers (Rizos and Elisaf, 2013). Wang et al. showed that in esophageal cancer, low doses of metformin reprogrammed the tumor immune microenvironment (TIME) in an anti-cancer direction by increasing the proportion of CD8^+^ cells (Wang et al., 2020). The combination of aloin and metformin was found to inhibit the growth and invasion of liver cancer through the PI3K/AKT/mTOR pathway and induce apoptosis and autophagy, thereby exerting anti-tumor effects (Sun et al., 2020). In addition, metformin and dichloroacetate inhibited the proliferation of liver cancer cells by inhibiting mTOR complex 1. Methotrexate is an anti-folate antitumor agent that inhibits the growth and multiplication of tumor cells by hindering their synthesis, mainly through the inhibition of dihydrofolate reductase (Rosh, 2022). Folic acid deficiency was found to cause defects in oxidative phosphorylation in human cells due to impaired mitochondrial translation, and thus affect energy metabolism (Morscher et al., 2018). Methotrexate has also been used as a therapeutic agent against lymphoma, osteosarcoma, and many other tumor types (Jaffe, 2009; Gaynon et al., 2010). In this study, patients with low EM scores showed higher sensitivity to the three chemotherapeutic agents mentioned above, suggesting that treatment with these three chemotherapeutic agents may be effective for patients with this type of liver cancer.

Kimiteru et al. showed that a CLTA-4 inhibitor (ipilimumab) was less effective in melanoma patients in a hypermetabolic state, and lead to shorter survival after treatment (Ito et al., 2018). In non-small cell lung cancer, patients with a larger total metabolic tumor volume (TMTV) have a shorter survival time after receiving immune checkpoint inhibitors (Seban et al., 2020). This suggests that EM scores may be related to the efficacy of immune checkpoint inhibitors in liver cancer treatment. Therefore, we analyzed the differences in sensitivity to PD-1 and CTLA-4 inhibitors between patients in the high and low EM score groups based on relevant data within the TCIA database. We found that patients in the low EM score group had higher sensitivity to PD-1 inhibitors, CTLA-4 inhibitors, and PD-1 inhibitors combined with CTLA4 inhibitors. Hence, immune checkpoint inhibition therapy may provide with a treatment option for patients with high EM scores.

## Conculsion

In this study, we identified a class of liver cancer cells with high energy metabolism from single-cell sequencing data, and determined five key genes related to energy metabolism on marker genes of this class of liver cancer cells combined with bulk sequencing data. We then constructed an EM score based on expression levels of these five genes to predict patient survival. In addition, we found that these five genes may be related to the sensitivity of liver cancer patients to chemotherapeutic drugs and immune checkpoint inhibitors. On the other hand, our findings are based on the exploration of public databases, and experimental validation is necessary. Moreover, specific mechanisms of regulation of energy metabolism in hepatocellular carcinoma cells by these signature genes need to be explored as well.

## Data Availability

The datasets presented in this study can be found in online repositories. The names of the repository/repositories and accession number(s) can be found in the article/Supplementary Material.

## Abbreviations

CDF, cumulative distribution function; CTLA-4, cytotoxic T-lymphocyte–associated antigen 4; EM Score, energy metabolism score; GEO, gene expression omnibus; HBV, hepatitis B virus; PD-1, programmed cell death protein 1; PD-L1, programmed death-ligand 1; ssGSEA, single-sample gene set enrichment analysis; SPC, sum of PCI; TCGA, the cancer genome atlas; TCIA, the cancer immunome atlas; TML, tumor mutation load; IARC, international agency for research on cancer; scRNA-seq: single-cell RNA sequencing data; TIME, tumor immune microenvironment; TMTV, total metabolic tumor volume.
