# Supplementary figures and images for: Identification of Energy Metabolism-Related Gene Signatures From scRNA-Seq Data to Predict the Prognosis of Liver Cancer Patients
Source: Front Cell Dev Biol. 2022 May 4;10:858336. doi: 10.3389/fcell.2022.858336 (PMC9114438; doi:10.3389/fcell.2022.858336)

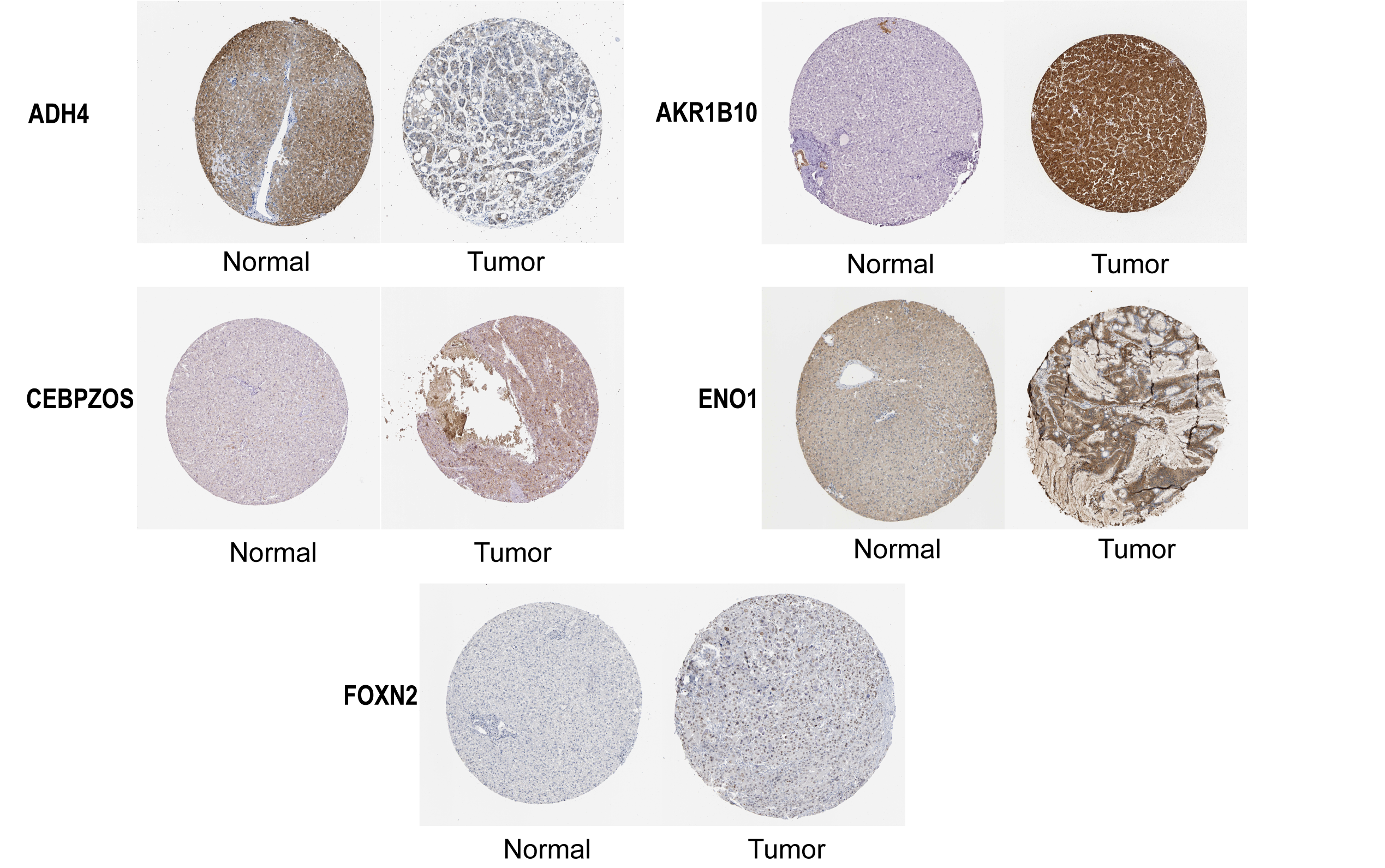

Supplement: Supplementary file 2 [file Image3.TIF]

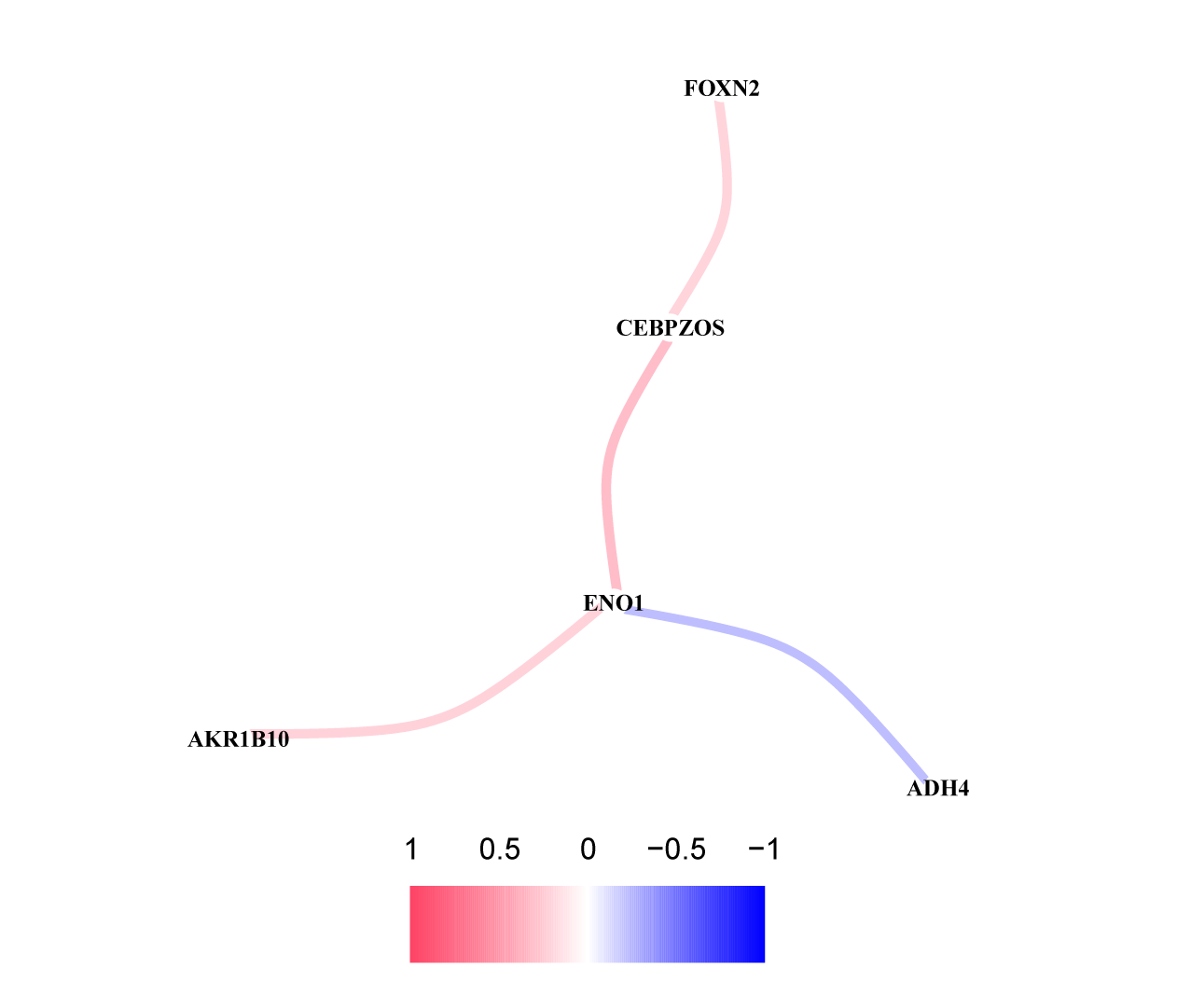

Supplement: Supplementary file 3 [file Image2.TIF]

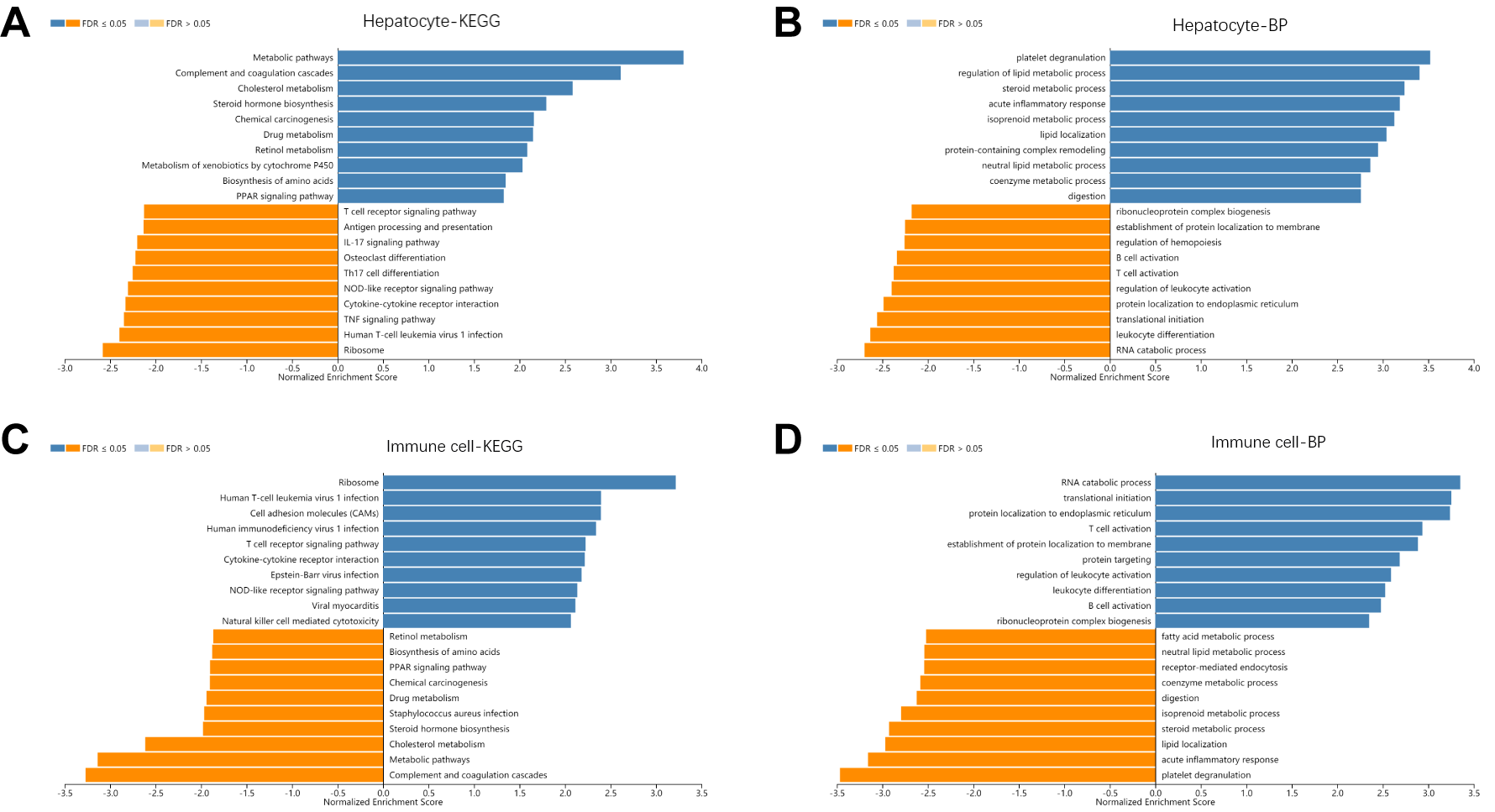

Supplement: Supplementary file 4 [file Image1.TIF]
