# Supplementary material for: Identification of Energy Metabolism-Related Gene Signatures From scRNA-Seq Data to Predict the Prognosis of Liver Cancer Patients
Source: Front Cell Dev Biol. 2022 May 4;10:858336. doi: 10.3389/fcell.2022.858336 (PMC9114438; doi:10.3389/fcell.2022.858336)
Supplement: Supplementary file 5 [file Table3.DOCX]

**Table S4: Metabolic pathways**

| **Metabolic pathway** | **PathwayID** | **Gene Count** |
| --- | --- | --- |
| Biological oxidations | R-HSA-211859 | 221 |
| Citric acid cycle (TCA cycle) | R-HSA-71403 | 22 |
| Glucose metabolism | R-HSA-70326 | 92 |
| Glycogen breakdown (glycogenolysis) | R-HSA-70221 | 15 |
| Glycogen metabolism | R-HSA-8982491 | 27 |
| Glycogen synthesis | R-HSA-3322077 | 16 |
| Glycolysis | R-HSA-70171 | 72 |
| Metabolism of carbohydrates | R-HSA-71387 | 292 |
| Mitochondrial Fatty Acid Beta-Oxidation | R-HSA-77289 | 38 |
| Pyruvate metabolism | R-HSA-70268 | 31 |
| Pyruvate metabolism and Citric Acid (TCA) cycle | R-HSA-71406 | 55 |
