# Supplementary material for: Identification of Energy Metabolism-Related Gene Signatures From scRNA-Seq Data to Predict the Prognosis of Liver Cancer Patients
Source: Front Cell Dev Biol. 2022 May 4;10:858336. doi: 10.3389/fcell.2022.858336 (PMC9114438; doi:10.3389/fcell.2022.858336)
Supplement: Supplementary file 7 [file Table4.DOCX]

**Table S5: The correlation results among the five genes**

| ID | ADH4 | AKR1B10 | CEBPZOS | ENO1 | FOXN2 |
| --- | --- | --- | --- | --- | --- |
| ADH4 | 1 | -0.158462554 | -0.188768563 | -0.253611337 | -0.150012823 |
| AKR1B10 | -0.158462554 | 1 | 0.000946976 | 0.238295232 | 0.131081675 |
| CEBPZOS | -0.188768563 | 0.000946976 | 1 | 0.3478917 | 0.231532221 |
| ENO1 | -0.253611337 | 0.238295232 | 0.3478917 | 1 | 0.093475545 |
| FOXN2 | -0.150012823 | 0.131081675 | 0.231532221 | 0.093475545 | 1 |
